# Production of L-carnitine-enriched edible filamentous fungal biomass through submerged cultivation

**DOI:** 10.1080/21655979.2020.1863618

**Published:** 2021-01-15

**Authors:** Neda Rousta, Jorge A. Ferreira, Mohammad J. Taherzadeh

**Affiliations:** Swedish Centre for Resource Recovery, University of Borås, Borås, Sweden

**Keywords:** *Aspergillus oryzae*, bioactive compounds, edible filamentous fungi, functional food, L-carnitine, submerged cultivation

## Abstract

The edible filamentous fungi are hot candidate for future supply of functional food and feed with e.g. protein, essential amino acids, and compounds with immunostimulant activity. L-carnitine that plays a crucial role in energy metabolism represents a functional compound normally produced by Zygomycetes filamentous fungus *Rhizopus oligosporus* in solid-state fermentation. The present study provides the first insights on production of L-carnitine-enriched edible fungal biomass through submerged cultivation of several Ascomycetes and Zygomycetes including *Aspergillus oryzae*, *Neurospora intermedia*, *Rhizopus oryzae*, and *Rhizopus oligosporus*. *A. oryzae* with 3 mg L-carnitine yield per gram of fungal biomass, indicates great potential on production of this bioactive compound which is remarkably higher than the other tested fungi in this work and also previous studies. In addition to fungal strain, other factors such as cultivation time and presence of yeast extract were found to play a role. Further studies on submerged growth optimization of *A*. *oryzae* in both high-quality recipes and in medium based on low-value substrates are proposed in order to clarify its potential for production of L-carnitine-enriched fungal biomass.

## 1. Introduction

With improving living standards, food conception is changing considerably [1]. Today, food is not regarded only as a source of energy to achieve hunger satisfaction but also acts as a factor for enhanced health benefits and reduced risks of disease [2]. Hence, the goal of nutrition science has been identifying foods that improve health beyond basic nutrition [1]. In this regard, bioactive compounds and food with biofunctionality play a remarkable role. One of the valuable sources of bioactive compounds is the fungal biomass of filamentous fungi [3]. Various types of natural products with bioactive properties can be found in filamentous fungal biomass [3,4] such as polyunsaturated fatty acids, vitamins [5], pigments [4], and heteropolysaccharides such as β-glucan [6].

One further bioactive compound that filamentous fungi are able to produce is L-carnitine. L-carnitine is a quaternary ammonium substance that plays a crucial role in energy metabolism. This compound acts as a shuttling molecule across the mitochondrial membrane [7] and transferring long-chain fatty acids for subsequent β-oxidation. According to its functionality, the effect of L-carnitine on weightloss and promoting physical performance has been reported [8–11]. Moreover, L-carnitine minimizes age-related disorders caused by free radicals due to its effective antioxidant activity [12–15]. Therefore, different applications such as in pharmaceutical and food sectors can be envisaged for L-carnitine [16].

As a result of its biofunctionality, L-carnitine has received attention in the supplement market over the last two decades. The market value of L-carnitine has reached USD 300 million in 2020 and it is projected to reach USD 338 million by 2026 [17]. The increasing commercial demand for either as pure compound or as carnitine-enriched food has led to the development of new production methods [18,19]. Most of the L-carnitine requirement in mammals is obtained by exogenous sources from animal products; only 25% is endogenously biosynthesized from two essential amino acids, namely, lysine and methionine [12,20]. Therefore, alternative L-carnitine sources are needed where food-grade filamentous fungi can become an important contributor. So far, all studies regarding L-carnitine production by filamentous fungi are based on solid-state fermentation for nutritional valorization of low-value solid residuals, leading to a heterogeneous final product composed of fungal mycelium and unconsumed substrate’s components [21,22]. Although solid-state fermentation is a valuable and low-energy intensive strategy for valorization of solid residuals, its scale-up is difficult and it is presently limited to small-scale applications for the production of human food products [23]. Most of the industrial processes employing filamentous fungi are carried out under submerged cultivation which benefits from a wide range of reactor designs, easier scale-up, and extensive industrial experience [24]. Inversely, submerged cultivation is characterized by higher energy demands. Therefore, it is important to investigate and develop L-carnitine production through submerged cultivation and provide a comprehensive basis for an efficient comparison with solid-state fermentation. However, no research studies are available on L-carnitine production from submerged cultivation of filamentous fungi.

This study aimed to investigate the effect of fungal strain, cultivation time, and medium composition on the production of L-carnitine by edible filamentous fungi under submerged cultivation. This work is a pioneer in the use of submerged cultivation for the production of this functional compound using filamentous fungi.

## 2. Materials and methods

### 2.1. Filamentous fungal strains

The edible fungal strains *Aspergillus oryzae* CBS 819.72, *Neurospora intermedia* CBS 131.92, *Rhizopus oligosporus* CBS 112586 (Centraalbureau voor Schimmelcultures, Utrecht, The Netherlands), and *Rhizopus oryzae* CCUG 61.147 (Culture Collection University of Gothenburg, Sweden) were used in the current study. The fungal strains were grown on potato dextrose agar (PDA) plates containing 20 g/l glucose, 15 g/l agar, and 4 g/l potato infusion. New PDA plates were prepared by flooding pre-grown plates with 20 ml of sterile distilled water; spores were brought into solution by using an L-shaped disposable plastic spreader. The new plates were then inoculated with 100 μl of spore solution, which was evenly spread onto the agar surface with a similar plastic spreader, followed by 3–5 days of incubation at 30°C and storage at 4°C until use [25].

### 2.2. Cultivation in shake flasks

The filamentous fungal strains were cultivated in 250-ml wide-necked cotton-plugged Erlenmeyer flasks containing 100 ml of the semi-synthetic medium. The medium was composed of 30 g/l of glucose, 5 g/l yeast extract, 4.55 g/l NaNO_3_, 3.5 g/l KH_2_PO_4_, 1 g/l CaCl_2_.2H_2_O, 2.25 g/l MgSO_4_.7H_2_O, 3.33 g/l lysine, 1.6 g/l methionine, and 10 ml/l trace metals solution and 1 ml/l vitamin solution according to Sues et al. [26]. The salt, amino acid, and glucose/yeast extract solutions were autoclaved separately at 121°C for 20 min. After adjusting the pH to 5.5 with a solution of 6 N HCl, 2 ml of spore solution was added to each flask followed by incubation in a water bath with orbital shaking of 125 rpm and at a temperature of 35°C [27]. The spore concentration for *A. oryzae, R. oryzae, N. intermedia, and R. oligosporus* were 12 × 10^5^, 10.5 × 10^5^, 6.7 × 10^5^, and 8.7 × 10^5^ spores/ml, respectively.

The fungal biomass was harvested from the medium at different time intervals (24, 48, and 72 h) using a stainless-steel kitchen sieve (1 mm^2^ pore area). Samples were taken from the remaining liquids and stored at −20°C for further analysis by high-performance liquid chromatography (HPLC) [27]. The biomass was thoroughly washed with distilled water to remove extracellular medium residuals followed by freeze-drying at 0.05 bar and −50°C to constant weight. Biomass mass concentrations are reported in grams of biomass per liter of the semi-synthetic medium. The dried cells were pulverized and prepared for L-carnitine analysis. All experiments were carried out in duplicate.

### 2.3. Analytical methods

For HPLC analysis, the liquid fractions of the medium were melted and centrifuged for 15 min at 20,000 × *g*; then, the supernatant was separated and filtered through a syringe filter (0.2 μm pore size, Sartorius). Analysis of glucose, acetic acid, ethanol, glycerol, and lactic acid, as components of the liquid part of fungal cultivation, was done using a hydrogen-ion-based ion-exchange column (Aminex HPX-87 H, Bio-Rad, Hercules, CA, USA) at 60°C and using 0.6 ml/min of 5 mM H_2_SO_4_ as eluent. The HPLC system was composed of Waters alliance separation module 2695 (Waters Corporation, Milford, MA, USA) coupled to a refractive index detector (Waters 2414) [25].

L-carnitine in the biomass was analyzed using an L-carnitine colorimetric and fluorometric assay kit (L-carnitine assay kit, Sigma-Aldrich) and identified by a NanoDrop spectrophotometer (Thermo Fisher Scientific, Wilmington, NC, USA). The concentration of L-carnitine was determined by a coupled enzyme assay. During the assay, the transfer of an acetyl group from coenzyme A (CoA) to carnitine takes place and the free CoA formed is further processed with subsequent oxidation of the OxiRed probe to give absorbance at 570 nm.

The crude protein content of the biomass was measured using the Kjeldahl method according to Mahboubi et al. [28]. The nitrogen-to-protein conversion was carried out by using a factor of 6.25.

The spore concentration of fungal spore solutions was quantified using a Bürker counting chamber under a light microscope (Carl Zeiss Axiostar Plus, Germany) [28].

### 2.4. Statistical analysis

All experiments were carried out in duplicate, and the statistical analysis of data was performed using the software MINITAB® 17 (Minitab Ltd., Coventry, UK). The error bars and intervals reported in the text, tables, and graphs represent two times the standard deviation. Analysis of variance was carried out using general linear models, and the values were considered statistically significant at *p*-value <0.05.

## 3. Results and discussion

**Figure 1.**
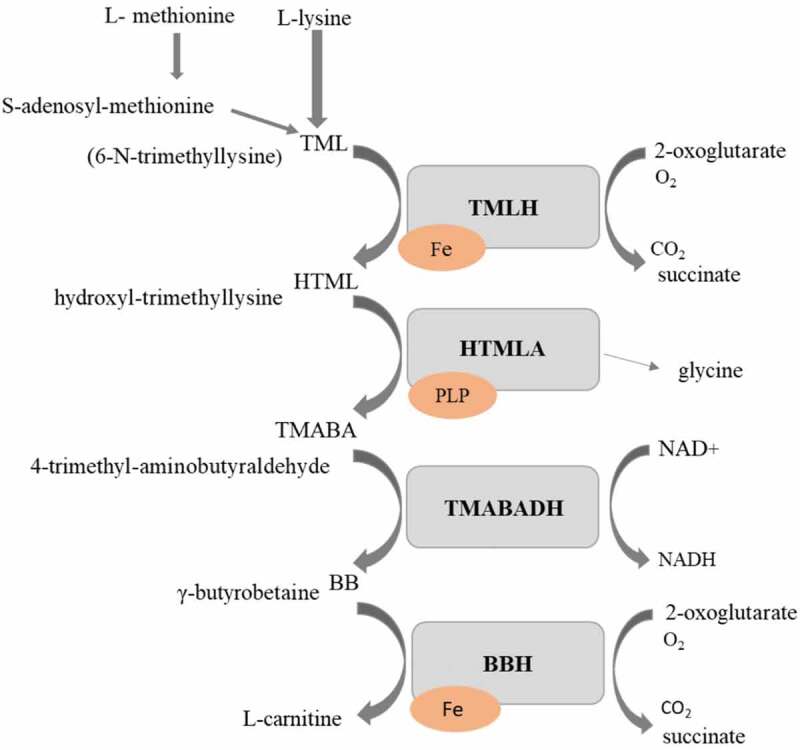
Carnitine biosynthesis pathway in fungi

For successful growth on different types of carbon sources, filamentous fungi need acetyl units. However, these cannot pass over the biological membrane freely. Therefore, L-carnitine is produced (Figure 1) in order to function as an acetyl unit transporter [29]. L-carnitine is synthesized from trimethyllysine following four enzymatic reactions. In the first step, L-lysine is methylated to trimethyllysine by methyltransferase that uses *S*-adenosyl methionine as a methyl donor. This reaction is followed by hydroxylation of trimethyllysine to hydroxyl-trimethyllysine (HTML) by the trimethyllysine hydroxylase. For this reaction, 2-oxoglutarate, oxygen, and Fe^2+^ are required as cofactors. 4-trimethyl-aminobutyraldehyde (TMABA) and glycine are formed through aldolytic cleavage of HTML by hydroxyl-trimethyllysine aldolase (HTMLA). This enzyme is dependent on pyridoxal 5′-phosphate as a cofactor. TMABA is dehydrogenated by the NAD^+^-dependent trimethyl-aminobutyraldehyde dehydrogenase, to form γ-butyrobetaine (BB). In the last step, butyrobetaine hydroxylase (BBH) hydroxylates BB to make L-carnitine. BBH requires 2-oxoglutarate, molecular oxygen, and Fe^2+^ as cofactors similarly to TMLD [30]. In fungi, fatty acid β-oxidation is entirely peroxisomal. After import into the peroxisome, acyl-CoA is oxidized completely to acetyl-CoA units. The carnitine shuttle, acetyltransferases, transfers the CoA group of acetyl-CoA to carnitine; hence, acetyl-carnitine is produced, which can be transported over the peroxisomal and mitochondrial membrane to the tricarboxylic acid cycle for adenosine triphosphate generation [30]. Similarly, the function of acetyl transportation by L-carnitine over the membranes is maintained during fungal growth in other sources of acetyl such as acetic acid, ethanol, and citric acid [30].

Presently, investigations on L-carnitine production are limited to solid-state fermentation. Albeit its low-energy character, the scale-up is challenging, and therefore it might be difficult to use for the production of bulk value-added products. Additionally, the final product of solid-state fermentation is normally a mixture of fungal biomass and undegraded medium compounds with a negative impact on the final concentration of the desired products. Submerged cultivation is comparatively more energy intensive but easier to scale up and it is presently responsible for most commercial products derived from filamentous fungi. Submerged cultivation of filamentous fungi offers the possibility of producing L-carnitine-containing fungal biomass with a lower effect of undegraded medium compounds on its composition in comparison to solid-state fermentation. Therefore, a final product with higher contents of protein, L-carnitine, among other compounds can be obtained. Additionally, due to their macroscopic growth, biomass originated through the cultivation of filamentous fungi can easily be recovered by low-energy strategies such as sieving. A variety of food-grade low-value substrates such as apple pomace or brewery spent grain find narrow applications in food systems due to low protein content and high amount of fibers [31–33]. Such a reality can be changed if the amount of protein is considerably increased. Filamentous fungi have extensively been used under both solid-state fermentation and submerged cultivation for the nutritional upgrade of a variety of low-value substrates [23]. However, studies on the production of L-carnitine are limited to solid-state fermentation, and therefore, studies under submerged cultivation are needed and should focus, in a first approach, on laboratory-made cultivation recipes preceding the transfer to medium based on low-value substrates. While developing filamentous fungi-based processes, cultivation factors such as strain, medium recipe, and physicochemical parameters are among the most commonly studied. However, specific and somewhat uncommon factors need also to be considered. For instance, lysine and methionine are two essential amino acids for L-carnitine production; lysine is the backbone of the carnitine molecule, while methionine acts as a methyl donor [34]. Altogether, four factors, namely, fungal strain, cultivation time, yeast extract addition, and ratio of lysine/methionine, on L-carnitine production were investigated in this work using a semisynthetic medium recipe.

### 3.1. Effect of fungal strain on L-carnitine production

In order to investigate the ability of edible filamentous fungi for L-carnitine production in submerged cultivation, four fungal strains were grown in a semisynthetic medium. All strains are food-grade and are traditionally used for the production of a variety of human fermented foods. Among the four strains, *A. oryzae* showed the greatest potential for L-carnitine production in submerged cultivation. Significant differences were found among groups of data, where *A. oryzae* was found to lead to the highest value based on pairwise comparisons (*p* = 0.001).

The amount of L-carnitine in *A. oryzae* pure biomass was three times, two times, and seven times and a half times higher than that found in the biomass of *R. oryzae, R. oligosporus*, and *N. intermedia*, respectively (Table 1). In addition to be limited to studies applying solid-state fermentation, a narrow range of strains, where *R. oligosporus* is the filamentous fungus of choice in single or in association with *Pleurotus ostreatus*, as well as a narrow range of substrates, namely buckwheat, quinoa, and ginseng, have been used for the production of L-carnitine [21,22,35,36]. Fermentation with *R. oligosporus* led to an increase of four times (from 168.6 to 680.9 μg/kg) in L-carnitine concentration of buckwheat [21]. The yolk of Hy-Line brown hens that were fed with fermented buckwheat as feed additive showed enriched L-carnitine (13.6%) in comparison with that of the control group [21]. A two-stage fermentation on buckwheat, including the first fermentation with *R. oligosporus* followed by fermentation with oyster mushroom (*Pleurotus ostreatus*), was found beneficial and led to the highest yield of L-carnitine, namely, of 201.2 mg/kg in comparison to that obtained following fermentation with only the mushroom (186.3 mg/kg) [22]. Further studies using *R. oligosporus* reported a yield of L-carnitine of 3.14 and 630 mg/kg following 3 days of fermentation on quinoa [36] and 14 days on wild ginseng [35], respectively (Table 2).

**Table 1.** L-carnitine concentrations, in milligrams per gram of fungal biomass following a 48-h cultivation of the zygomycetes and ascomycetes strains in a semisynthetic medium

Fungal strain	L-carnitine (mg/g dried biomass)
*A. oryzae*	3 ± 0.9
*R. oryzae*	1.3 ± 0.4
*R. oligosporus*	1 ± 0.6
*N. intermedia*	0.4 ± 0.04

**Table 2.** The data comparison on L-carnitine production by filamentous fungi in the present study with previous studies

Type of microorganism	Kind of substrate	Final result of L-carnitine production	Time of fermentation	Type of fermentation	References
*A. oryzae*	Semisynthetic medium	3 mg/g dried biomass	48 h	Submerged cultivation	This work
*R. oryzae*		1.3 mg/g dried biomass			This work
*R. oligosporus*		1 mg/g dried biomass			This work
*N. intermedia*		0.4 mg/g dried biomass			This work
*R. oligosporus*	wild ginseng	630 mg/kg	14 days	Solid-state fermentation	[37]
*R. oligosporus,+ Pleurotus ostreatus*	Buck wheat	201.2 mg/kg	-		[22]
*R. oligosporus*	Quinoa	3.14 mg/kg	3 days		[38]
*R. oligosporus*	Buck wheat	Increasing from 168.6 μg/kg to 680.9 μg/kg	5–7 days		[21]

Therefore, the present study introduces new strains, namely, *A. oryzae* and *R. oryzae,* as potential L-carnitine producers considering the yield of L-carnitine found in their biomass in comparison to that found in the biomass of *R. oligosporus*. Naturally, these results also motivate further screening of other food-grade zygomycetes and ascomycetes filamentous fungi.

### 3.2. Effect of cultivation time on L-carnitine production

**Figure 2.**
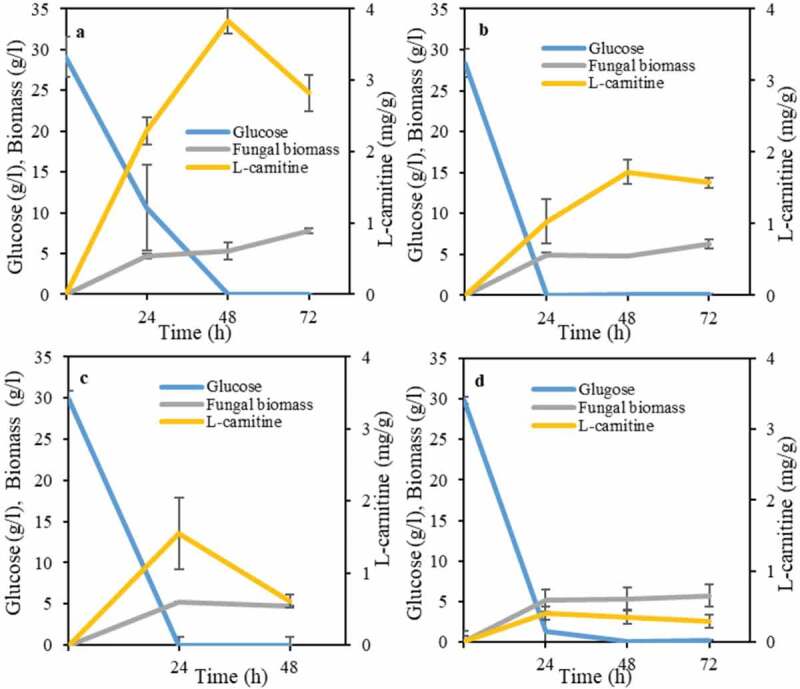
Profiles of L-carnitine yields, glucose consumption and biomass mass concentration during growth of ascomycetes and zygomycetes filamentous fungi, namely, (a) *A. oryzae* , (b) *R. oryzae*, (c) *R. oligosporus*, and (d) *N. intermedia* in the semisynthetic medium

A more detailed study was carried out in order to investigate the effect of cultivation time on L-carnitine production. There was a direct relationship between glucose availability in the medium with L-carnitine production for three strains, namely, *A. oryzae, N. intermedia*, and *R. oligosporus* (Figure 2). In other words, there was an increasing trend in L-carnitine production until glucose is depleted in the medium, where a decreasing trend followed. In principle, it seems that L-carnitine acts as a primary product similarly to other microbial products such as ethanol. Total glucose consumption and the highest L-carnitine yield, namely of 3.8 mg per gram of biomass, through cultivation with *A. oryzae* occurred after 48 h (Figure 2). For *N. intermedia* and *R. oligosporus*, the highest levels of L-carnitine (0.4 and 1.5 mg per gram of biomass, respectively) were obtained after 24 h of cultivation coinciding with glucose depletion (Figure 2). Only *R. oryzae* showed different trends. Although the fungus consumed glucose completely after 24 h of cultivation, the increasing trend of L-carnitine continued up to 48 h and reached 1.7 mg per gram of biomass followed by a decreasing trend (Figure 2).

It seems that as long as glucose is present in the medium, it serves as the main source of energy and carbon, preventing L-carnitine consumption. However, after glucose depletion, and although the biomass weight kept increasing, the percentage of L-carnitine in biomass started to decrease. Therefore, most probably, L-carnitine was used as a carbon source by the filamentous fungal strains in the absence of glucose and points out the importance of cultivation medium optimization.

### 3.2. The effect of yeast extract on L-carnitine production

**Figure 3.**
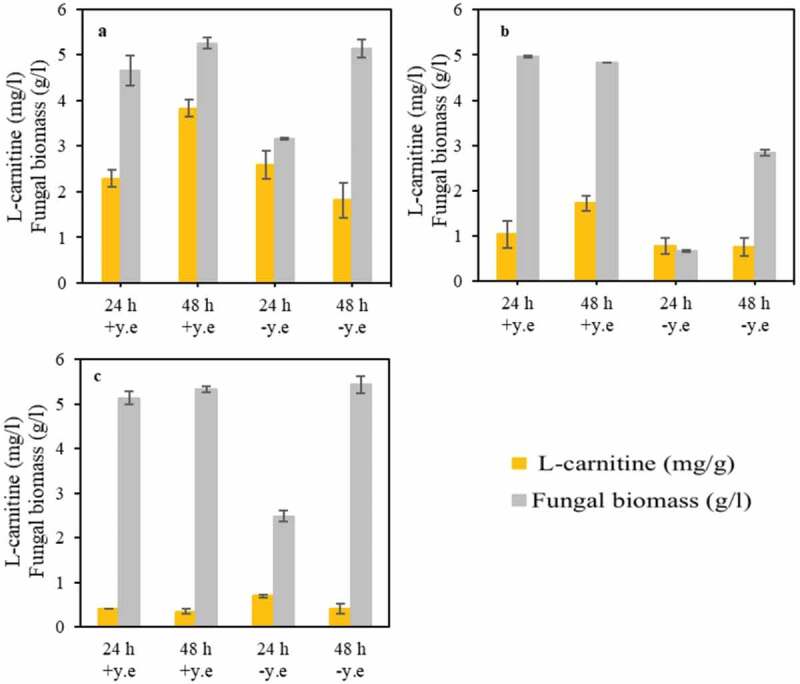
The effect of yeast extract presence and absence on fungal biomass concentration and yield of L-carnitine during cultivation of (a) *A. oryzae*, (b) *R. oryzae*, (c) and *N. intermedia*

Yeast extract is normally added to the medium to serve mostly as a nitrogen source. However, it is a mixture of several compounds including amino acids, and therefore its effect on L-carnitine production was investigated (Figure 3).

The effect of yeast extract on L-carnitine was dissimilar among the strains used. The amount of L-carnitine found in the biomass of *A. oryzae* in the presence of yeast extract was significantly higher than that achieved during *A. oryzae* cultivation in medium without yeast extract (Figure 3). Moreover, an increasing trend in L-carnitine production was observed when yeast extract was present in the medium, being the inverse observed in the absence of yeast extract. An increasing trend in L-carnitine concentration over cultivation time was also observed during the cultivation of *R. oryzae* in the presence of yeast extract. However, the absence of yeast extract did not influence L-carnitine production (Figure 3). A similar result was observed for *N. intermedia* in both the presence and absence of yeast extract pointing out that this fungus might not be a potential candidate for L-carnitine production. The use of the two ascomycetes (*A. oryzae* and *N. intermedia*) and two zygomycetes (*R. oryzae* and *R. oligosporus*) in this study was motivated by their dissimilar characteristics. For instance, the cell wall of zygomycetes is mainly composed of a mixture of chitin and chitosan, whereas that of ascomycetes is mainly based on chitin [37], to which immunostimulant activity has been ascertained [38]. Moreover, *N. intermedia* has been found to be a superior ethanol producer [39], providing a diversity of products that can potentially be obtained from the valorization of low-value substrates.

### 3.3. The effect of lysine/methionine ratio on L-carnitine production

**Figure 4.**
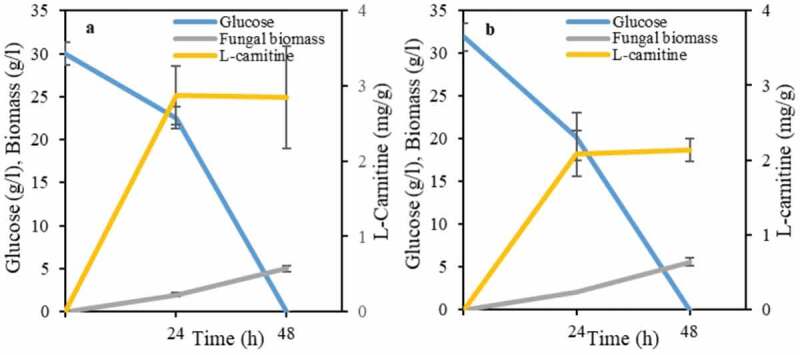
The effect of lysine/methionine ratio, namely, of (a) 2 and (b) 4 on L-carnitine yield during cultivation of *A. oryzae.*

During endogenous production of L-carnitine, lysine serves as the carbon skeleton and it is methylated with *S*-adenosyl-methionine derived from the amino acid methionine [34]. Accordingly, the effect of two ratios, namely, 2 and 4, of lysine/methionine on L-carnitine production during cultivation of *A. oryzae*, the best L-carnitine producer found in this study, was investigated. The medium recipe used for the experiments reported in the previous sections contained a ratio of lysine/methionine of 2. A higher lysine/methionine ratio present in the cultivation medium does not have a positive effect on L-carnitine production (Figure 4), where a concentration of 33% lower was obtained.

### 3.4. Fungal biomass protein and L-carnitine contents

Forecasts of insufficient animal protein sources as well as environmental issues led to a focus on microbial production of protein to meet human requirements. The fungal biomass of filamentous fungi is characterized by high-protein contents which is a positive starting point for food applications [40]. On industrial scale, mycoprotein from *Fusarium venenatum* with 44% protein content has become well admitted as a suitable, high-protein, microbial substitute for human consumption [41].

**Figure 5.**
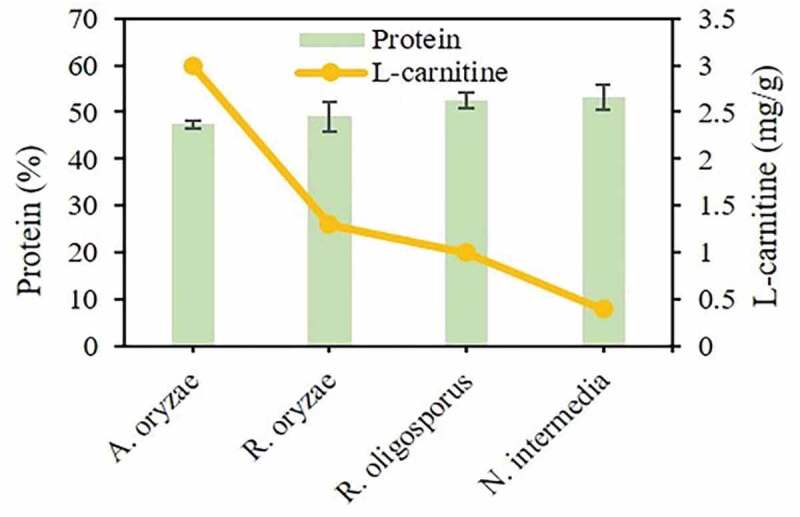
Profiles of protein and L-carnitine contents in the fungal biomass originated from submerged cultivation of the filamentous fungal strains used in this study

Enriching the biomass with other bioactive compounds such as L-carnitine increases the value and relevance of fungal biomass. The protein content of all fungal biomasses produced in this study contained around 50% protein on a dry weight basis (Figure 5). Therefore, *A. oryzae* is proposed in this study as a potential source of bioactive compounds in view of comparable protein content and the highest amount of L-carnitine found among the strains investigated.

Several studies in the literature [27,39,42–44] have reported protein contents in the fungal biomass comparable to those obtained in this study, while many others reported much lower protein contents in view of entanglement of suspended solids with fungal filaments during submerged cultivation [45–47]. On the other hand, the protein content of the mixture of fungal biomass and unconsumed substrate’s compounds following solid-state fermentation will naturally be based on the dominance of fungal biomass. Both strategies need, however, optimization of various parameters such as medium composition, nutrient supplementation, cultivation time, choice of strain, and overall physicochemical parameters (e.g., temperature, pH) for proper fungal growth. From a low-value substrate valorization point-of-view, solid-state fermentation can represent a suitable strategy for small-scale processes dealing with small quantities of substrates, whereas submerged cultivation can have a higher impact on the amount of valorized substrate. Through literature survey and comparison (Table 2), the results from this study point out that submerged cultivation can attain higher productivities of L-carnitine-enriched fungal biomass in comparison to that achieved through solid-state fermentation. Therefore, it is hypothesized that further efforts should be laid down on the development of submerged cultivation processes for production of L-carnitine-enriched fungal biomass. However, both solid-state fermentation and submerged cultivation have both superficially been investigated, and therefore, it is immature to state the most advantageous technique for production of L-carnitine-enriched fungal biomass with potential feed and food applications. In addition to cultivation optimization and process scale-up using different substrates, the research on L-carnitine production needs also to be linked to in vitro digestion simulation studies in order to clarify its functional effects.

**Figure:**
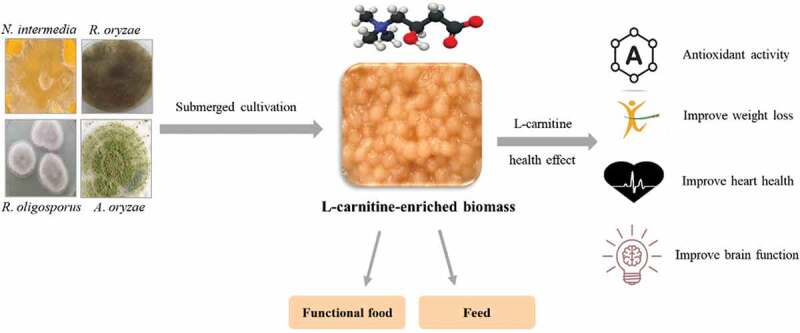

## 4. Conclusions

Filamentous fungi can have a high contribution to the production of alternative functional feed and food products in view of their composition rich in a wide range of functional compounds and versatility of cultivation systems they can be integrated into. In this study, the ability of four edible filamentous fungi, namely, *A. oryzae, R. oryzae, R. oligosporus,* and *N. intermedia*, were screened to produce L-carnitine in a semisynthetic medium. Solid-state fermentation has been the strategy of choice for production of L-carnitine, while this work introduces, for the first time, the use of submerged cultivation and discusses its advantages and potentialities. *A. oryzae* was found to be a potential producer of this functional product, which is applicable in both feed and food systems. On the other hand, the ascomycete *N. intermedia*, a fungus of high interest for the development of biotechnological processes toward ethanol production, was found to be a fungus with comparatively much lower potential for production of L-carnitine-enriched fungal biomass. Further studies on process optimization and transfer of knowledge to cultivation medium containing low-value substrates need to be carried out in order to clarify the potential of *A. oryzae* for the production of L-carnitine-enriched fungal biomass through submerged cultivation. This should parallelly be developed with *in vitro* digestion studies to unveil the functional value of the newly produced L-carnitine-enriched fungal biomass.

## Supplementary Material

Supplemental Material

## Article highlights

- L-carnitine-enriched edible fungal biomass was produced by submerged cultivation.
- This nutritious pure fungal biomass can be used as functional food and feed.
- *A.Oryzae* represented the great capacity for L-carnitine production.
