# Supplementary material for: Production of L-carnitine-enriched edible filamentous fungal biomass through submerged cultivation
Source: Bioengineered. 2021 Jan 15;12(1):358–68. doi: 10.1080/21655979.2020.1863618 (PMC8806343; doi:10.1080/21655979.2020.1863618)
Supplement: Supplemental Material [file KBIE_A_1863618_SM4985.zip › supplement/Highlights.docx]

Highlights

- L-carnitine-enriched edible fungal biomass was produced by submerge cultivation.
- This nutritious pure fungal biomass can be used as functional food and feed.
- *A. oryzae* represented the great capacity on L-carnitine production.
