# Supplementary figures and images for: Production of L-carnitine-enriched edible filamentous fungal biomass through submerged cultivation
Source: Bioengineered. 2021 Jan 15;12(1):358–68. doi: 10.1080/21655979.2020.1863618 (PMC8806343; doi:10.1080/21655979.2020.1863618)

## Slide 1
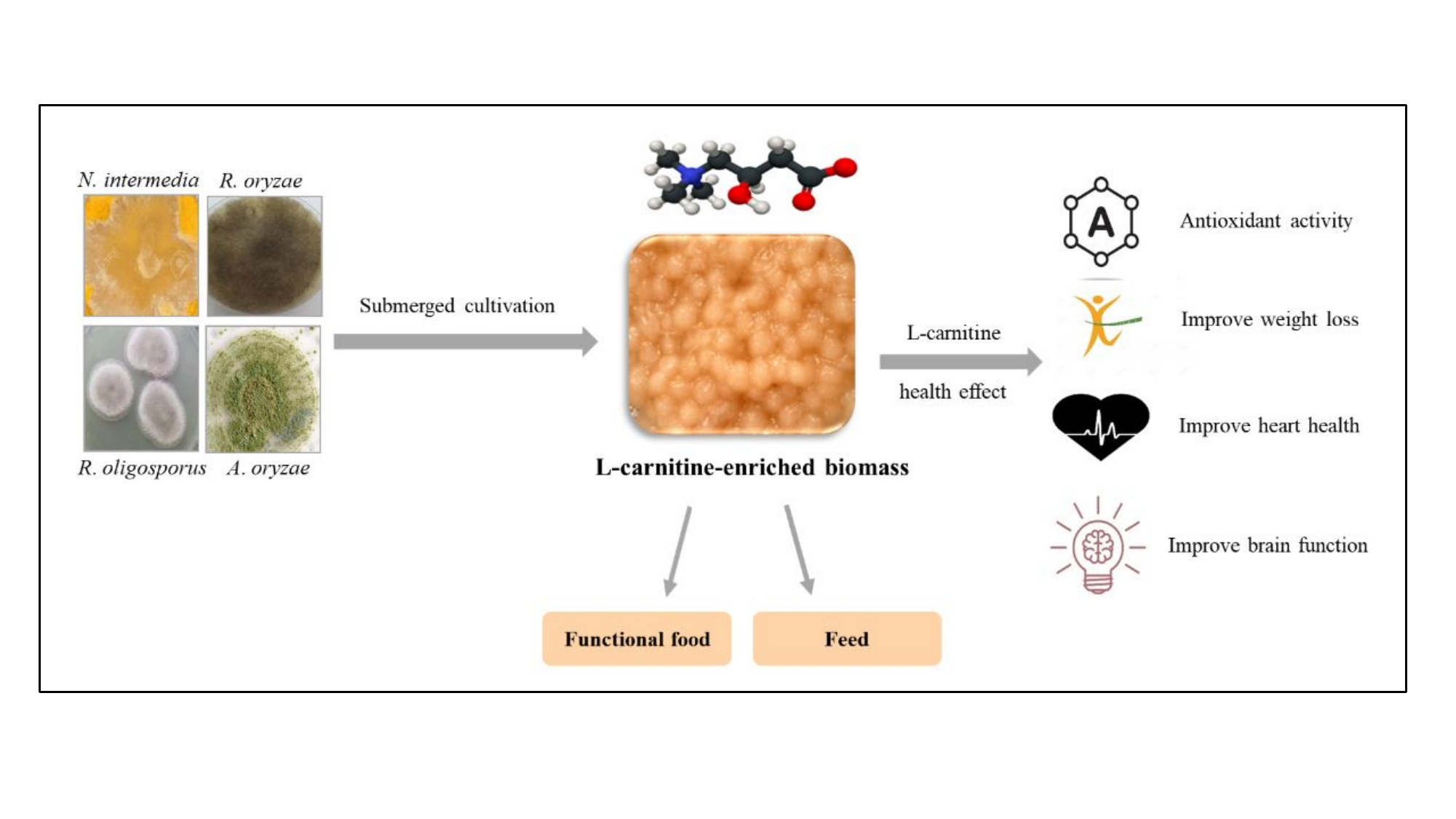

## Slide 2
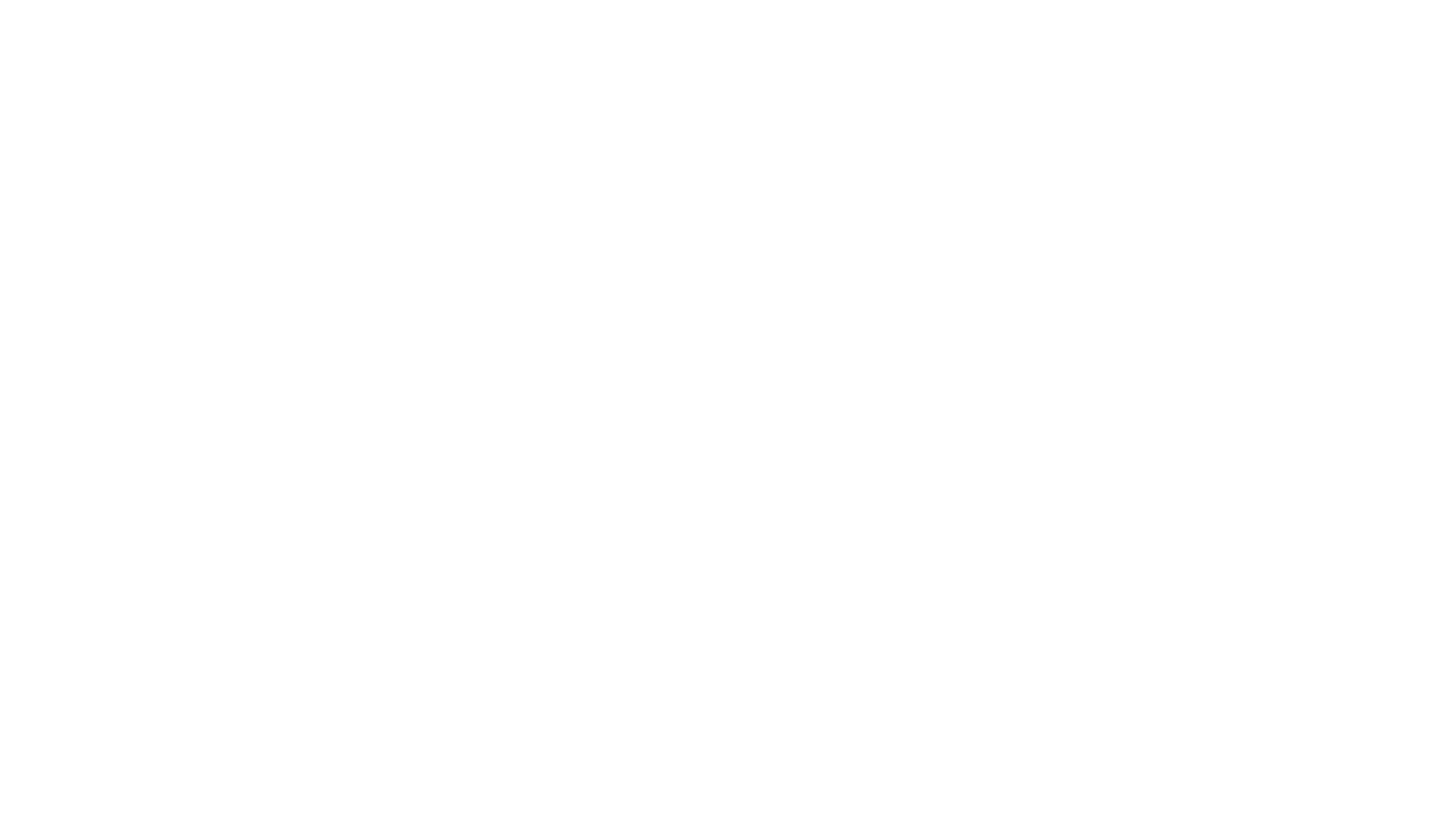

Supplement: Supplemental Material [file KBIE_A_1863618_SM4985.zip › supplement/GraphicalAbstractR1.pptx]
